# A rapid and robust method for the cryopreservation of human granulosa cells

**DOI:** 10.1007/s00418-021-02019-3

**Published:** 2021-07-27

**Authors:** Sarah Beschta, Katja Eubler, Nancy Bohne, Ignasi Forne, Dieter Berg, Ulrike Berg, Artur Mayerhofer

**Affiliations:** 1 Cell Biology, Anatomy III, Faculty of Medicine, Biomedical Center Munich (BMC), Ludwig-Maximilian-University (LMU), 82152 Martinsried, Germany; 2 Fertility Centre A.R.T. Bogenhausen, 81675 Munich, Germany; 3 Protein Analysis Unit, Faculty of Medicine, Biomedical Center Munich (BMC), Ludwig-Maximilian-University (LMU), 82152 Martinsried, Germany

**Keywords:** Cryopreservation, IVF, Ovarian cells, Cell culture, Mass spectrometry, Progesterone

## Abstract

Human primary granulosa cells (GCs) derived from women undergoing oocyte retrieval can be cultured and used as a cellular model for the study of human ovarian function. In vitro, they change rapidly, initially resembling cells of the preovulatory follicle and then cells of the corpus luteum. They are derived from individual patients, whose different medical history, lifestyle and age lead to heterogeneity. Thus, cells can rarely be ideally matched for cellular experiments or, if available, only in small quantities. We reasoned that cryopreservation of human GCs may be helpful to improve this situation. Previous studies indicated the feasibility of such an approach, but low survival of human GCs was reported, and effects on human GC functionality were only partially evaluated. We tested a slow freezing protocol (employing FCS and DMSO) for human GCs upon isolation from follicular fluid. We compared cryopreserved and subsequently thawed cells with fresh, non-cryopreserved cells from the same patients. About 80% of human GCs survived freezing/thawing. No differences were found in cell morphology, survival rate in culture, or transcript levels of mitochondrial (*COX4*, *OPA1, TOMM20*), steroidogenic (*CYP11A1*, *CYP19A1)* or cell–cell contact genes (*GJA1*) between the two groups in cells cultured for 1–5 days. A proteomic analysis revealed no statistically significant change in the abundance of a total of 5962 proteins. The two groups produced comparable basal levels of progesterone and responded similarly to hCG with elevation of progesterone. Taken together, our results show this to be a rapid and readily available method for the cryopreservation of human GCs. We anticipate that it will allow future large-scale experiments and may thereby improve cellular studies with human ovarian cells.

**Supplementary Information:**

The online version contains supplementary material available at 10.1007/s00418-021-02019-3.

## Introduction

The development and widespread use of assisted reproductive technology (ART) has opened a unique possibility for the study of the human ovary, namely follicular fluid and granulosa cells. Human granulosa cells (GCs) are the by-product of follicular aspiration performed on women undergoing medical procedures like in vitro fertilization (IVF), intracytoplasmic sperm injection (ICSI) or cryopreservation of their oocytes. They are therefore readily available and represent, in general, mural cells, which are in the process of luteinization (luteinizing GCs). They can be isolated from the follicular fluid, cultured in vitro and then studied (Blohberger et al. 2016; Bulling et al. 2000; Saller et al. 2014). As GCs fulfill an essential role in the follicle, form the environment for the oocyte and produce steroids, the study of cultured human GCs provides a unique window into the human ovary. A previous study employing a proteomic comparison revealed that IVF-derived human GCs in culture resemble initially luteinizing cells of the ovulatory follicle, but then are more comparable to the large luteinized granulosa cells of the corpus luteum (CL) (Bagnjuk and Mayerhofer 2019). During the culture period, human GCs further undergo striking changes and at later time points they are alike the cells of the regressing CL (Bagnjuk and Mayerhofer 2019). Thus, cultured human GCs are highly dynamic and provide insight into different situations of the human ovary.

Indeed, the changes are rapid and occur within a few days; hence human GCs rarely can be ideally matched for cellular experiments. The cells stem from humans and due to patients’ age, lifestyle and their medical history, human GCs are notoriously heterogeneous, a situation aggravated in conditions such as polycystic ovarian syndrome (PCOS). We reasoned that upon cryopreservation of human GCs, sufficient numbers of comparable cells may become available to improve cellular studies.

There are several studies demonstrating cryopreservation of ovarian tissues and cells in various species, including human and bovine (Amorim et al. 2011; Baufeld and Vanselow 2018; Bouillon et al. 2020; Kokotsaki et al. 2018; Pietrowski et al. 2020; Rivas Leonel et al. 2019; Santana et al. 2012; Shi et al. 2017; Sluss et al. 1994; Youm et al. 2014; Zheng et al. 2019). They indicate that freezing/thawing is, in general, possible, yet the survival rate of cells is rather low (45–58%; see Bouillon et al. 2020). Furthermore, the consequences of such procedures for GCs are not fully evaluated, especially for IVF-derived human GCs (Bouillon et al. 2020; Sluss et al. 1994).

Here we report a method for cryopreservation and later use of human GCs and show by employing different approaches, including a proteomic analysis, that the cryopreserved cells do not differ from their fresh counterparts.

## Materials and methods

### Human GC isolation and cell culture

For the isolation of human GCs, follicular fluid was used from patients undergoing medical procedures including in vitro fertilization (IVF), intracytoplasmic sperm injection (ICSI) or cryopreservation of oocytes primarily to address infertility. Patients with polycystic ovary syndrome (PCOS) were excluded from this study. By written consent, patients agreed to the scientific use of biological material, as part of ongoing projects within the framework of a German Research Foundation (DFG)-funded project (456828204). The use of human cells was approved by the Ethics Committee of LMU (Project number 20-697). Follicular fluid from two to six patients (average 3.2 ± 0.3 punctures) was pooled for this study, and human GCs were isolated using a cell strainer, as described previously (Blohberger et al. 2016). After centrifugation of the cell suspension for 3 min at 800 rpm, isolated cells were mixed with trypan blue (Lonza, Basel, Switzerland) and counted using a Neubauer chamber.

The resulting number of cells was equally divided and one half was used for cryopreservation and the other half was seeded (1–1.25 × 10^5^ cells/dish) onto p35 cell culture dishes (Sarstedt, Leicester, UK) containing Dulbecco's modified Eagle medium/Ham’s F-12 nutrient mixture (DMEM/F12; Gibco, Paisley, UK) supplemented with 1% penicillin/streptomycin (P/S; BioChrom, Berlin, Germany) and 10% fetal calf serum (FCS; Capricorn Scientific, Ebsdorfergrund, Germany) and kept at 37 °C, 5% CO_2_ and 95% humidity until experimental use. On the first day of culture, human GCs were washed thoroughly with pure DMEM/F12 to get rid of remaining blood cells and tissue fragments and fresh supplemented culture medium (DMEM/F12 with 1% P/S and 10% FCS) was added. Cells were cultured up to 5 days for this study and medium was changed every other day.

### Cryopreservation and thawing

For cryopreservation, half of the freshly isolated cells (422,375 ± 57,910 cells, *n* = 16) were resuspended in 1 ml DMEM/F12 containing 1% P/S, 10% FCS and 10% dimethyl sulfoxide (DMSO; Sigma-Aldrich, St. Louis, MO, USA) and were transferred to a cryotube (Thermo Fisher Scientific, Waltham, MA, USA). Cells were cooled to −80 °C at a rate of −1 °C/min using an alcohol-free cell freezing container (BioCision CoolCell 1 ml FX, Brooks Life Sciences GmbH, Griesheim, Germany) and were transferred to liquid nitrogen the following day. Cells tested in this study were stored for up to 14 days in a liquid nitrogen tank. On the day of use, cells were rapidly thawed in a water bath at 37 °C and transferred to a falcon tube with about 7 ml DMEM/F12 supplemented with 1% P/S and 10% FCS. The suspension was then centrifuged for 3 min at 800 rpm and the pellet was resuspended in fresh supplemented culture medium. To determine the survival rate after this freeze/thaw process, cells were mixed with trypan blue and recounted using a Neubauer chamber. Cells, in identical numbers to those of freshly isolated human GCs, were plated on p35 cell culture dishes, washed thoroughly with pure DMEM/F12 on culture day 1 and kept at 37 °C, 5% CO_2_ and 95% humidity until experimental use. Cells were cultured up to 5 days for this study, and the medium was changed every other day.

### RNA isolation and reverse transcriptase quantitative PCR (RT-qPCR)

Cultured human GCs were washed thoroughly with 1 ml phosphate-buffered saline (PBS; Thermo Fisher Scientific) and RNA was isolated using the RNeasy Plus Micro Kit (Qiagen, Hilden, Germany) following the manufacturer’s instructions. Depending on the amount of RNA, reverse transcription was performed with 400 ng to 1 µg RNA using random 15-mer primers (Metabion, Planegg, Germany) and SuperScript II (Invitrogen, Carlsbad, CA, USA). Water within the reaction instead of enzyme served as a non-reverse transcription control, RT-qPCR was performed with 4 ng cDNA in the reaction, and RNA instead of cDNA was used as negative control using the QuantiFast SYBR Green PCR Kit (Qiagen). The mRNA levels of the investigated genes were set in relation to the housekeeper *RPL19* [ratio: *C*_q_ (*RPL19*) / *C*_q_ (gene of interest)], compared between the coherent fresh and frozen/thawed samples [ratio (frozen/thawed) / ratio (fresh)], and are expressed as the difference between the thawed and fresh samples [△: 1 − ratio (frozen/thawed) / ratio (fresh)]. Detailed information about the oligonucleotide primers is depicted in Table 1.

**Table 1:** Oligonucleotide primer sequences, annealing temperatures, amplicon sizes and references

Gene	Sequence	Annealing temperature (°C)	Amplicon size (bp)	GenBank ID
*COX4*	5′-AGC GAG CAA TTT CCA CCT CT-3′ 5′-TCA CGC CGA TCC ATA TAA GCT-3′	59	90	NM_001318797.1
*CYP11A1*	5′-TCG GCA GCC TGG AAG AAA GAC C-3′ 5′-GGC GCT CCC CAA AAA TGA CG-3′	59	226	NM_001099773.2
*CYP19A1*	5′-GCT ACC CAG TGA AAA AGG GGA-3′ 5′-GCC AAA TGG CTG AAA GTA CCT AT-3′	59	140	NM_000103
*GJA1*	5′-CAA TCA CTT GGC GTG ACT TC-3′ 5′-CCT CCA GCA GTT GAG TAG GC-3′	60	120	NM_000165
*OPA1*	5′-CTC TGC AGG CTC GTC TCA AG-3′ 5′-CAC ACT GTT CTT GGG TCC GA-3′	60	108	NM_130831.2
*RPL19*	5′-AGG CAC ATG GGC ATA GGT AA-3′ 5′-CCA TGA GAA TCC GCT TGT TT-3′	59	199	NM_000981.3
*StAR*	5′-ACG TGG ATT AAC CAG GTT CG-3′ 5′-CAG CCC TCT TGG TTG CTA AG-3′	58	149	NM_000349
*TOMM20*	5′-CCC CAA CTT CAA GAA CAG GC-3′ 5′-GAT GGT CTA CGC CCT TCT CA-3′	60	185	NM_014765.3

### Proteomics

Proteomic analysis was performed on culture day 3 with fresh and frozen/thawed human GCs from three individual batches. In brief, human GCs (1 × 105) were detached from the plate using Trypsin–EDTA solution (L2143, Biochrom GmbH, Berlin, Germany), and the reaction was stopped with 1.5 ml DMEM/F12 supplemented with 1% P/S and 10% FCS. The cells were washed three times with 1 ml PBS, and after the third washing step the excess liquid was removed and the cell pellet was frozen at −80 °C. Samples were processed using the PreOmics iST sample preparation kit (PreOmics GmbH, Planegg/Martinsried, Germany) as recommended by the manufacturer.

For liquid chromatography–mass spectrometry (LC–MS) purposes, desalted peptides were injected in a nanoElute system (Bruker, Billerica, MA, USA) and separated in a 25-cm analytical column (75 µm ID, 1.6 µm C18, IonOpticks) with a 100-min gradient from 2 to 37% acetonitrile in 0.1% formic acid. The effluent from the high-performance (HP)LC was directly electrosprayed into a hybrid trapped ion mobility-quadrupole time-of-flight mass spectrometer (timsTOF Pro, Bruker Daltonics, Bremen, Germany) using the nano-electrospray ion source at 1.4 kV (CaptiveSpray, Bruker Daltonics). The timsTOF was operated at 100% duty cycle in data-dependent mode to automatically switch between one full TIMS-MS scan and ten PASEF [parallel accumulation–serial fragmentation] MS/MS scans in a range from 100 to 1700 m/z in positive electrospray mode with an overall acquisition cycle of 1.23 s. The ion mobility was scanned from 0.6 to 1.60 Vs/cm^2^ with TIMS ion charge control set to 5e4, RF potential of 300 Vpp. The TIMS dimension was calibrated linearly using four selected ions from the Agilent ESI LC/MS tuning mix [m/z, 1/K0: (322.0481, 0.7318 Vs/cm^2^), (622.0289, 0.9848 Vs/cm^2^), (922.0097, 1.1895 Vs/cm^2^), (1221.9906, 1.3820 Vs/cm^2^)] (Agilent Technologies, Inc., Santa Clara, CA, USA). MaxQuant 1.6.10.43 was used for protein identification and label-free quantitation (LFQ) with the following parameters: Database, Uniprot_AUP000005640_Hsapiens_20200120.fasta; MS tol, 10 ppm; MS/MS tol, 20 ppm Da; Peptide false discovery rate (FDR), 0.1; Protein FDR, 0.01 Min. peptide length, 7; Variable modifications, oxidation (M); Fixed modifications, carbamidomethyl (C); Peptides for protein quantitation, razor and unique; Min. peptides, 1; Min. ratio count, 2. Identified proteins were considered as differential if their log2 fold change (LFQ) values were higher than log2. The list of all proteins detected is provided in the supplementary data (S1). The data were analyzed with Perseus software (Computation Systems Biochemistry, Martinsried, Germany). The mass spectrometry proteomics data were deposited to the ProteomeXchange Consortium via the PRIDE (Proteomics Identifications Database) partner repository with the dataset identifier PXD025632.

### Progesterone measurements

On culture day 3, fresh and frozen/thawed human GCs (*n* = 5 each) were starved for 2 h in colorless DMEM/F12 without any supplements and then treated with 10 IU/ml human chorionic gonadotropin (hCG; Sigma-Aldrich) or corresponding solvent control (0.01 M NaH_2_PO_4_, Sigma-Aldrich), respectively, for 24 h in the same medium. Supernatant was collected and stored at −20 °C for measurement of progesterone content using the IMMULITE 2000 XPi immunoassay system (Siemens Healthineers, Erlangen, Germany). The quality of the measurement is being assessed regularly, as this system is used for routine clinical measurements. The inter-assay and intra-assay coefficients of variation are 0.059 and 0.036, respectively.

Cells were washed with cold PBS and protein was isolated using RIPA buffer supplemented with protease and phosphatase inhibitors (Thermo Fisher Scientific). Total protein amount was determined via the Lowry assay (DCTM Protein Assay; Bio-Rad Laboratories, Inc., Hercules, CA, USA) by measuring the absorbance values at 690 nm (FLUOstar OPTIMA, BMG LABTECH GmbH, Ortenberg, Germany) and interpolating the individual samples to a defined standard curve of bovine serum albumin (BSA, GE Healthcare, Solingen, Germany) dissolved in RIPA buffer ranging from 0 to 1.5 µg/µl, as described before (Blohberger et al. 2016). Protein values were used for normalization of measured progesterone levels.

### Data analysis and statistics

Microscopical images were captured using a Leica DM IL LED microscope (Leica Microsystems GmbH, Wetzlar, Germany), equipped with a ×10 objective (HI Plan CY ×10/0.25 dry, Leica Microsystems) and a monochrome camera (DFC3000 G, Leica Microsystems) with the corresponding software (Leica Applications Suite X, version 3.7.0.20979, Leica Microsystems). Pictures were brightness and contrast adjusted using Fiji (open source image processing package for imageJ).

RT-qPCR data sets and progesterone measurements were analyzed using Microsoft Excel (2018, Microsoft, Redmond, WA, USA), and statistical analysis was performed with GraphPad Prism 7 software (GraphPad Software, San Diego, CA, USA). Normal distribution was assessed (Shapiro–Wilk normality test, *α* = 0.05), and one-way analysis of variance (ANOVA) was used for comparison of relative mRNA expression levels of fresh and frozen/thawed human GCs. A paired two-tailed *t*-test was used for comparison of progesterone levels and cell survival rates after 3 days in culture of fresh and frozen/thawed human GCs. α was set to 0.05 (*p < 0.05, **p < 0.01, ***p < 0.001), and data are depicted as mean ± SEM.

## Results

### Cell survival rate and gene expression

**Fig. 1:**
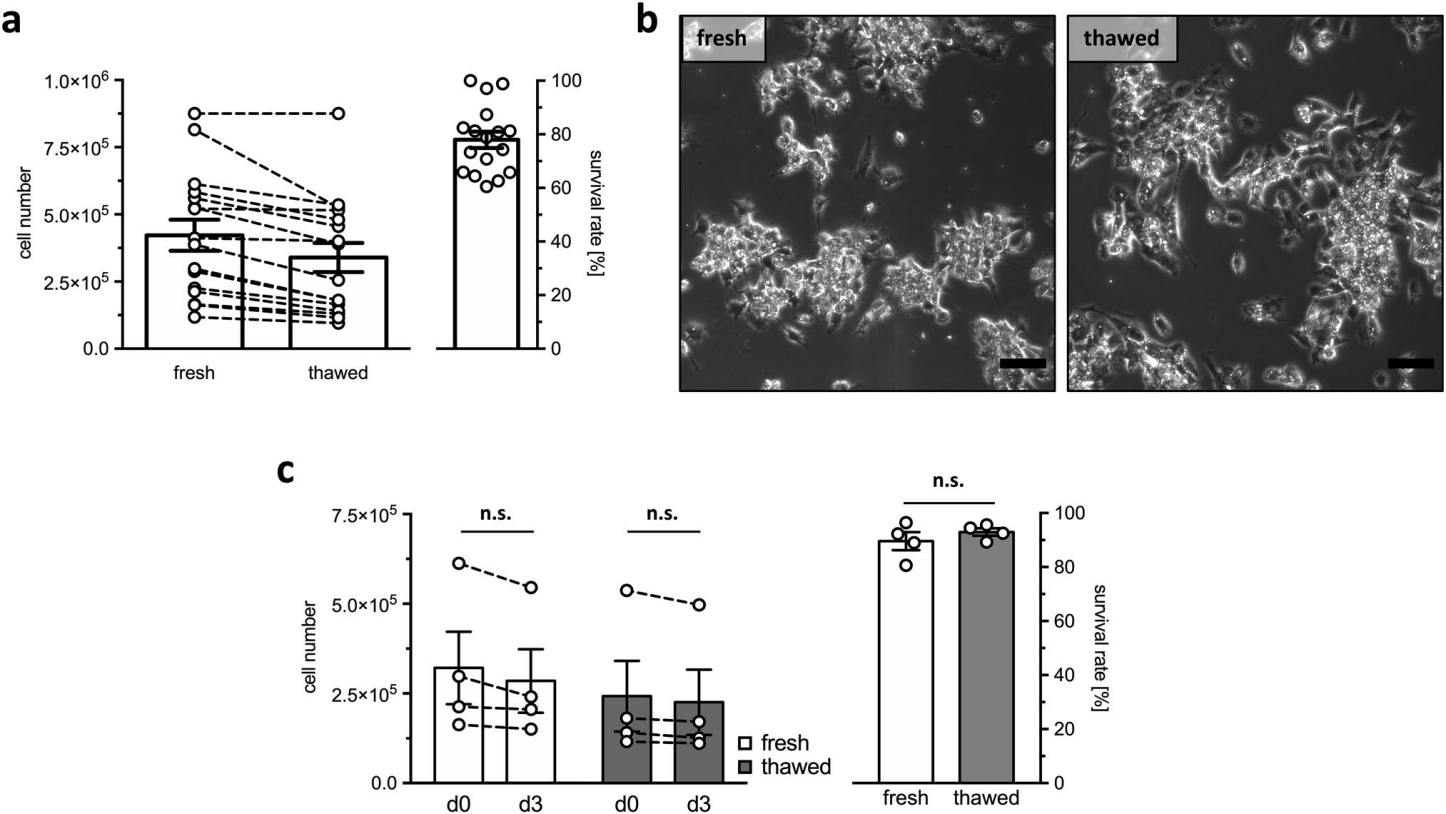
Cell numbers, survival rate and morphological appearance of fresh and frozen/thawed human GCs. **a** Cell numbers of fresh and frozen/thawed cells (left panel) and survival rate (%) of human GCs after freezing and thawing (right panel), depicted as mean ± SEM (*n* = 16 each). **b** Phase-contrast images of fresh (left) and frozen/thawed (right) human GCs on culture day 3. No difference in morphological appearance of the cells was observed and both fresh and frozen/thawed cells showed typical clustering. Scale bar 100 µm. **c** Cell numbers of fresh (white bars) and frozen/thawed cells (grey bars) on culture days 0 and 3, depicted as mean ± SEM (*n* = 4 each), and values of individual batches, with dashed lines representing coherent counts (left panel). Survival rate (%) of fresh (white bar) and frozen/thawed human GCs (grey bar) after 3 days in culture (right panel), depicted as mean ± SEM (*n* = 4 each). Upon given normal distribution of data sets (Shapiro–Wilk normality test, *α* = 0.05), both cell numbers and survival rates were statistically analyzed using a paired two-tailed *t*-test, *α* = 0.05; *n.s.* not significant

To examine, whether human GCs tolerate the freezing/thawing procedure, we counted the cells both directly after the isolation and after cryopreservation and thawing. The mean cell number directly after isolation amounted to 422,375 ± 57,910 counted cells and after cryopreservation and following thawing, a total of 339,688 ± 54,460 cells could be counted (*n* = 16 each). The results indicate that 77.8 ± 3.2% (*n* = 16) of human GCs survived the procedure of cryopreservation and thawing (Fig. 1a). Inspection by light microscopy indicated that the freezing and thawing process had no discernable influence on the general morphology (shape and size) of the cells. Both the fresh (left) and the frozen/thawed cells (right) showed typical clustering (Fig. 1b). To further investigate to which degree human GCs tolerate cryopreservation, corresponding fresh and frozen/thawed cells were counted after 3 days in culture (Fig. 1c). Freshly isolated and seeded human GCs exhibited a survival rate of 89.6 ± 3.3% after 3 days in culture, which is statistically indistinguishable from the survival rate of cryopreserved cells, at 93.0 ± 1.4% (*p* = 0.4885, *n* = 4).

**Fig. 2:**
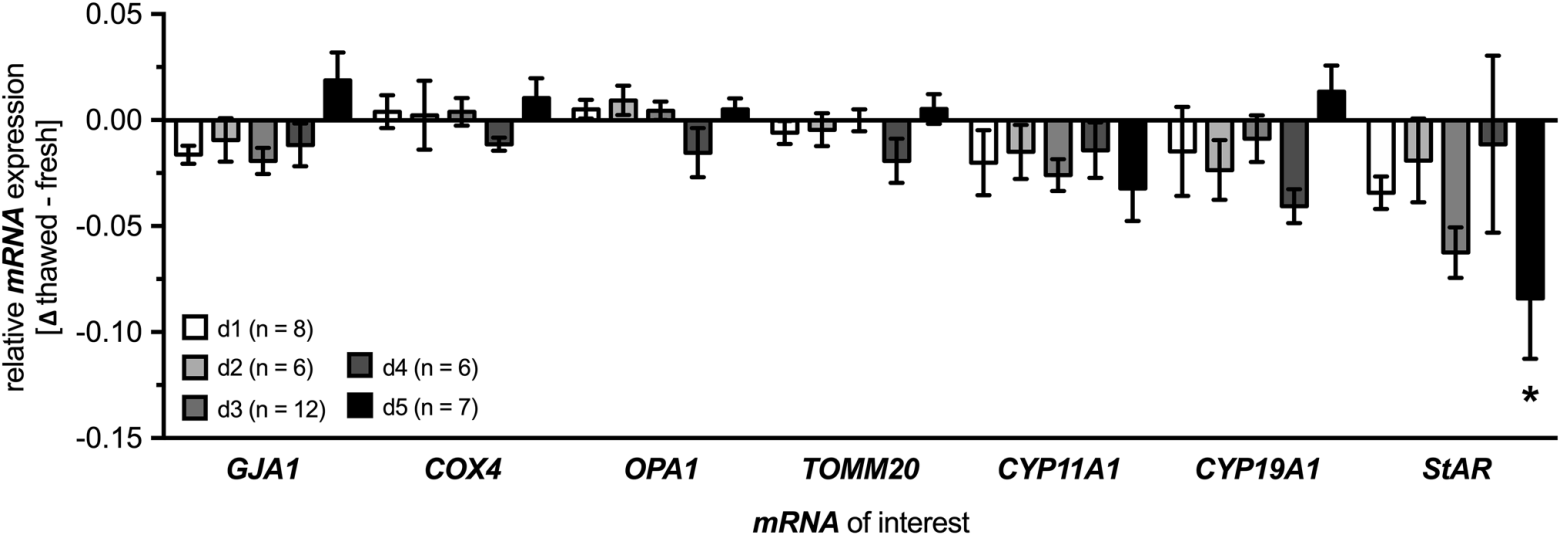
Relative mRNA expression levels in frozen/thawed human GCs. mRNA expression levels of marker genes for cell–cell contact (*GJA1*) and mitochondrial (*COX4*, *OPA1*, *TOMM20*) and steroidogenic function (*CYP11A1*, *CYP19A1*, *StAR*) in frozen/thawed human GCs compared to their coherent freshly isolated and seeded cells, harvested on cell culture days 1–5 (d1–5); mean ± SEM (*n* = 6–12). One-way ANOVA, *α *= 0.05; *p < 0.05

Next we isolated mRNA from freshly isolated and seeded human GCs and their frozen/thawed counterparts at different cell culture days (days 1–5) to examine key markers by RT-qPCR. We focused on gap junctional cell–cell contact mitochondrial and steroidogenic markers (Fig. 2). For both the analyzed cell–cell contact marker *GJA1*, essential for gap junction formation, and all investigated mitochondrial markers—i.e. *COX4*, an important component of the mitochondrial electron transport chain; *OPA1*, essential for mitochondrial fusion process; and *TOMM20*, required for recognition and translocation of mitochondrial pre-proteins across the outer mitochondrial membrane—slight variability could be observed, which did not reach statistical significance independently of the cell culture day studied. Regarding mRNA levels of three important steroidogenic genes—i.e. *CYP11A1*, catalyzing the cholesterol cleavage to pregnenolone; *CYP19A1*, catalyzing for conversion of androgens to estrogens; and *StAR*, essential for steroid hormone synthesis—the strongest differences were found in terms of reduced expression levels in the frozen/thawed human GCs. However, only the mRNA level of *StAR* at cell culture day 5 was slightly, yet statistically significantly, lower (−0.084 ± 0.03, *p* = 0.026) compared to corresponding coeval freshly isolated and seeded cells.

### Proteomics data analysis

**Fig. 3:**
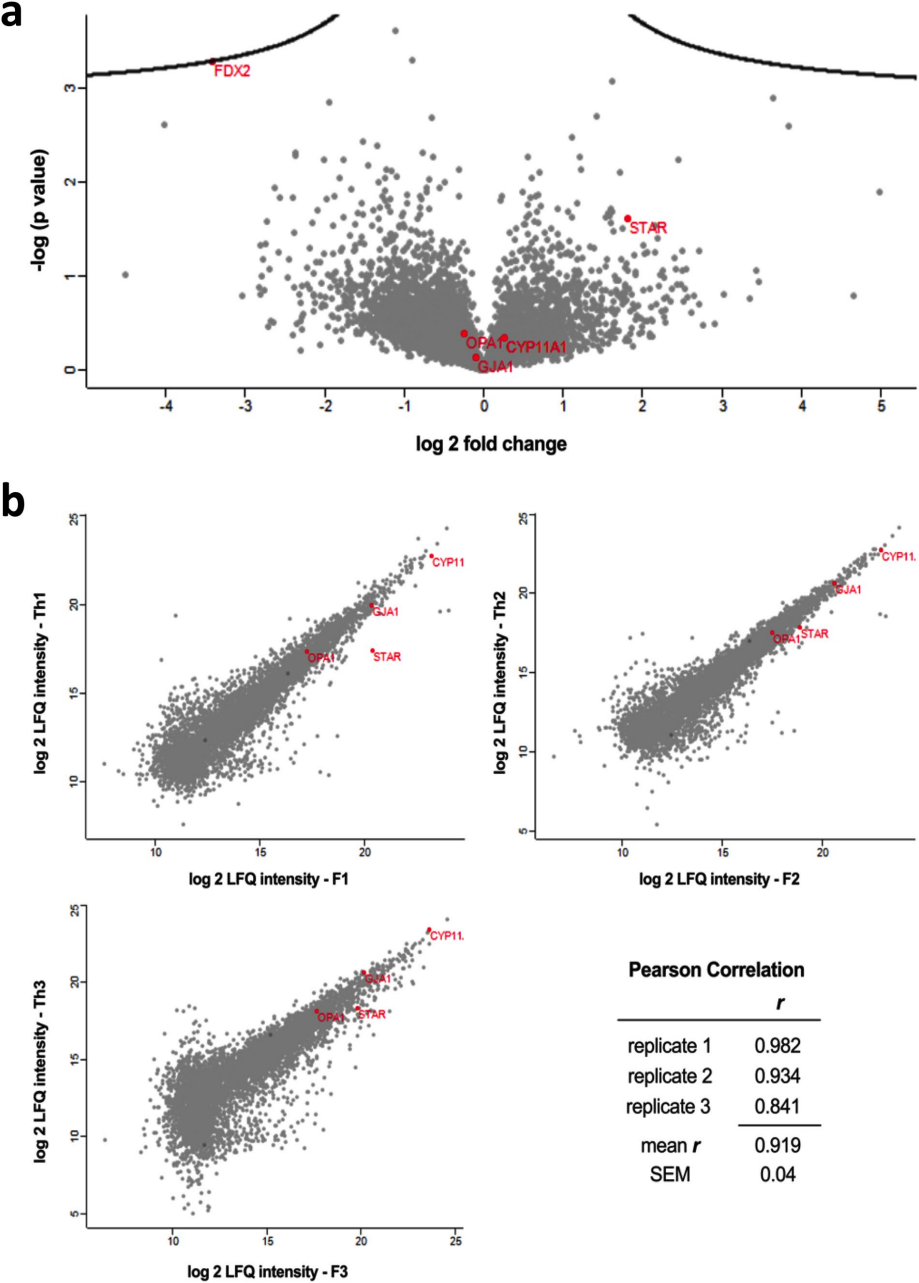
Proteomics data of freshly isolated and seeded human GCs and the corresponding frozen/thawed cells. **a** Volcano Plot of differentially regulated proteins in response to freezing and thawing. Fresh and frozen/thawed human GCs from culture day 3 were harvested and proteins were quantified by mass spectrometry. Proteins were plotted by difference (log twofold change) and significance (−log10 *p*-value) using a false discovery rate (FDR) of 0.45 and a minimal fold change (s0) of 0.1. No significant up- or downregulation was observed for the 5962 analyzed proteins using Perseus software. **b** Scatter Plots of relative protein amount in response to freezing and thawing from the three biological replicates. Log2 LFQ intensities of fresh (F) and frozen/thawed (Th) human GCs are compared, and the corresponding Pearson correlation coefficients are depicted, indicating a strong linear relation of the protein amount. Genes also analyzed by RT-qPCR are indicated in red, and the position of the FDX2 is given

To explore differences that may exist between freshly isolated human GCs and cryopreserved and subsequently thawed cells, a proteomics data analysis was performed. To this end, fresh and frozen/thawed cells of three biological replicates were harvested on cell culture day 3 and analyzed by means of mass spectrometry. In total, 5962 proteins were detected (see Supplementary data), and differences in protein levels between the fresh and frozen/thawed human GCs were visualized in a volcano plot (Fig. 3a). Even at very generous conditions, with a false discovery rate (FDR) of 0.45 and a minimal fold change (s0) of 0.1, no significant up- or downregulation was observed. Even ferredoxin 2 (FDX2), essential for heme A and iron-sulfur protein biosynthesis, which showed the largest aberrance, was within this range.

Furthermore, the individual biological replicates show very strong positive correlations, with a mean Pearson correlation coefficient *r* of 0.919 ± 0.04, distinguishable by the scatter plots shown in Fig. 3b.

### Progesterone levels

**Fig. 4:**
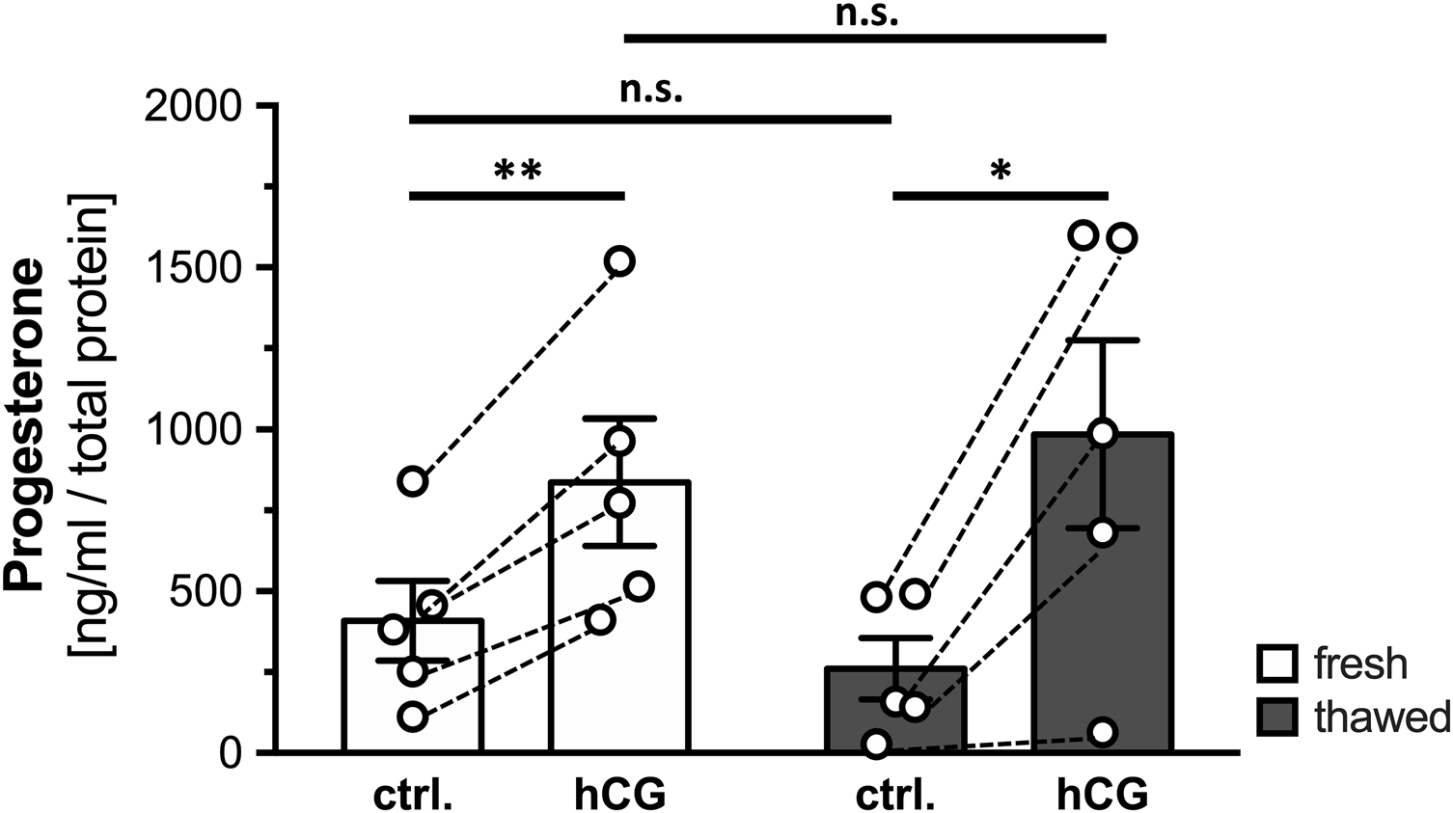
Progesterone levels in the supernatants from freshly isolated and seeded human GCs and the corresponding frozen/thawed cells. Progesterone levels in the supernatants from freshly isolated and seeded human GCs (white bars) and frozen/thawed cells (grey bars) after administration of 10 IU/ml hCG (hCG) and corresponding solvent control (ctrl.) for 24 h on cell culture day 3. Values are mean ± SEM (*n* = 5 each), coherent measurements are depicted by dashed lines. Paired two-tailed *t*-test, *α* = 0.05; *p < 0.05, **p < 0.01, *n.s.* not significant

To further examine the potential influence of freezing and thawing on the ability to produce progesterone, we studied this point on cell culture day 3. Both freshly isolated and seeded human GCs and frozen/thawed GCs (*n* = 5 each) were treated with 10 IU/ml hCG or corresponding solvent control for 24 h, and supernatants were examined for their progesterone content (Fig. 4).

Under control conditions, supernatants of freshly isolated and seeded human GCs contained 408.6 ± 123.0 ng/ml progesterone, statistically indistinguishable from the levels of supernatants from frozen/thawed cells with 260.5 ± 95.2 ng/ml progesterone under these conditions (*p* = 0.0901). Upon administration of hCG, the level of progesterone increased significantly in the supernatants from both groups (fresh: 836.9 ± 196.1 ng/ml, *p* = 0.007; frozen/thawed: 985.2 ± 290.2 ng/ml, *p* = 0.0228), compared to the corresponding control supernatants. The levels of progesterone in supernatants from freshly isolated and seeded human GCs and frozen/thawed cells upon hCG administration were similar (*p* = 0.4799).

## Discussion and conclusion

The research with primary, patient-derived human GCs holds promise for human-focused research, especially because human GCs in vitro are an apt model for the ovulatory follicle and the corpus luteum (Bagnjuk and Mayerhofer 2019). In practical terms, work with human GCs has limitations, particularly with respect to daily availability of comparable cells, given the source (i.e., individual patients) and the rapidly changing phenotype of cultured human GCs.

We reasoned that cryopreservation of human GCs may allow the collection of comparable cells over time, and thus may foster more precise, as well as large-scale experiments. We therefore tested whether a routine freezing method, used for cell lines in our laboratory, is also suitable for human GCs. We initially observed that human GCs tolerate the freezing/thawing procedure rather well and thus we performed a thorough investigation to examine consequences on cell composition and function.

Several freezing methods have been tested for ovarian tissue and its cells (Bouillon et al. 2020; Kokotsaki et al. 2018; Pietrowski et al. 2020; Rivas Leonel et al. 2019; Santana et al. 2012; Sluss et al. 1994), including human GCs and the human granulosa tumor cell line KGN. Cryopreservation by slowly freezing using a cryoprotectant and ultra-fast freezing by vitrification were tested, employing DMSO or ethylene glycol solutions (Kokotsaki et al. 2018). In these studies, usually only a few cell parameters were evaluated, with a focus on cell survival or cell death, respectively (Kokotsaki et al. 2018).

Sluss et al. and Bouillon et al. studied human GCs. In the more recent study (Bouillon et al. 2020), employing human GCs, two different freezing protocols were compared. The authors report that GCs tolerate both procedures, albeit with a rather poor survival rate of 45–58%, while survival of only about 32% was described in the older study by Sluss et al. (1994). The surviving cells remained responsive to follicle-stimulating hormone (FSH) stimulation after freezing/thawing, although FSH efficacy was decreased (Bouillon et al. 2020). The study compared freezing of cell pellets directly derived from follicular fluids, without further purification steps and freezing of cells after a Percoll purification step to remove red blood cells. In both cases, a solution containing 90% FCS and 10% DMSO and a concentration of 1 × 10^6^ cells/ml and vial were used. A detailed description of the actual freezing method is, however, not provided. Our present study tried to find a simple, readily available freezing method also involving FCS and DMSO, and hence we adopted a method used in our laboratory for cell lines, including KGN cells. The results indicated a loss of about 20% of the cells, which may be partly related to the cytotoxic effect of DMSO (Santos et al. 2003). The survival rate of about 80% is, however, tolerable from a practical point of view. It is comparable to the one reported for the human GC line KGN (Kokotsaki et al. 2018; Pietrowski et al. 2020), in which related slow freezing methods were employed, and it is much higher than the low survival rate reported by a recent study of human GCs (Bouillon et al. 2020).

Furthermore, we found that when cells were cultured for 3 days, the survival rate of freshly plated human GCs did not differ from that of their frozen/thawed counterparts.

To thoroughly examine further consequences of the method, we first performed RT-qPCR screening of key markers of human GCs, namely the major gap junction gene *GJA1*, mitochondrial and steroidogenic genes, along with mass spectrometry analysis. The RT-qPCR results revealed no significant differences in transcript levels of selected genes between the groups of fresh and frozen/thawed cells, except for *StAR*, which was slightly reduced in frozen/thawed cells, albeit only < 10% and only on culture day 5. Most likely this small change in mRNA abundance is not of biological relevance.

A thorough analysis of three batches of cells was performed on culture day 3, employing mass spectrometry. With this approach, we monitored almost 6000 proteins, and none of them differed in abundance between the two groups.

Further, we examined the ability of fresh and frozen/thawed cells to produce progesterone, the major steroid of the corpus luteum. No difference was found in either basal or hCG-stimulated production of this hormone. Hence, the frozen/thawed and the fresh cells are virtually indistinguishable from each other. Sluss et al. also measured steroids, i.e. basal production of estradiol and progesterone (Sluss et al. 1994). For cryopreserved cells, basal sex steroid secretion was reduced after cryopreservation (20% for estradiol and approximately 50% for progesterone), while aromatase activity was not different. While we did not examine estradiol syntheses directly, unchanged aromatase levels in the proteome analysis indicate that this function is fully retained. Furthermore, basal and stimulated progesterone production indicate that neither LH receptor signaling nor steroid machinery suffer from freezing/thawing.

In summary, we describe a simple, readily available method, which allows cryopreservation of human GCs upon isolation from follicular fluid. The human GCs retained their integrity and functionality. Furthermore, only 20% of the cells did not survive the procedure. The surviving 80% were functionally indistinguishable from fresh, non-cryopreserved human GCs. We anticipate that this method, which is superior to previously described methods, may facilitate future studies.

## Supplementary Information

Below is the link to the electronic supplementary material.

Supplementary file1 (XLSX 2621 KB)

## Data Availability

The mass spectrometry proteomics data have been deposited to the ProteomeXchange Consortium (https://www.ebi.ac.uk/pride/) via the PRIDE [1] partner repository with the dataset identifier PXD025632.
